# Unveiling Functional Architecture of Human Thalamus: AStereoelectroencephalography Study

**DOI:** 10.1002/mco2.70931

**Published:** 2026-09-02

**Authors:** Liankun Ren, Di Wu, Cuiping Xu, Lei Qi, Guangyuan Jin, Xueyuan Wang, Yueyang Cheng, Jialin Du, Siyi Wang, Dongmei Hu, Junliang Ge, Yixi Zheng, Yihe Wang, Huaqiang Zhang, Yang Dai, Hao Yan, Runshi Gao, Ying Gao, Zeyu Zhang, Penghu Wei, Yuping Wang, Philippe Kahane, Fabrice Bartolomei, Tao Yu, Guoguang Zhao

**Affiliations:** ^1^ Department of Neurology Xuanwu Hospital Capital Medical University Beijing China; ^2^ Clinical Research Center of Epilepsy Xuanwu Hospital Capital Medical University Beijing China; ^3^ National Center for Neurological Disorders Beijing China; ^4^ Department of Neurosurgery Xuanwu Hospital Capital Medical University Beijing China; ^5^ Department of Neurology China Rehabilitation Research Center Beijing China; ^6^ Department of Pharmacy Phase I Clinical Trial Center Xuanwu Hospital Capital Medical University Beijing China; ^7^ Department of Science The University of Melbourne Victoria Australia; ^8^ Institute of Sleep and Consciousness Disorders Center of Epilepsy Beijing Institute for Brain Disorders Capital Medical University Beijing China; ^9^ Grenoble Alpes University Hospital Center Grenoble Alpes University, Inserm, U1216 Grenoble Institute of Neurosciences Grenoble France; ^10^ Institut de Neurosciences des Systèmes Inserm, INS Aix‐Marseille University Marseille France; ^11^ Epilepsy and Clinical Neurophysiology Department Timone Hospital Assistance Publique‐Hôpitaux de Marseille Marseille France

**Keywords:** direct electrical stimulation, epilepsy, functional connectivity, thalamus

## Abstract

As a critical hub in the thalamo‐cortical circuit, the human thalamus engages in a spectrum of fundamental and advanced brain functions through widely distributed circuits. Clinically, dysfunction of thalamo‐cortical circuits is shown to be profoundly implicated in a wide range of neurological and psychiatric diseases. Nevertheless, the neuroanatomical substrates governing these functions of the thalamus have rarely been directly mapped in humans. Here, we overviewed the acute responses of direct electrical stimulation (DES) delivered to the distributed thalamus sites in 52 epilepsy patients admitted for presurgical stereoelectroencephalography. Specifically, DES of the thalamus evoked a broad spectrum of in‐situ responses spanning fundamental functions such as sensory and motor processing, extending to complex neural operations encompassing neurovegetative regulation, cognitive processing, emotional modulation, and multimodal responsiveness. Moreover, through the integration of DES with functional human connectome (*n* = 1000), we found an intra‐thalamic intrinsic functional network associated with each specific clinical response. Our data provide direct substantiation for the complex functional architecture of the human thalamus, advancing current understanding of its role and potentially culminating in targeted therapeutic strategies tailored to ameliorate symptom‐specific neural circuit disorders.

## Graphical abstract

The human thalamo‐cortical loop underpins complex behavior and is implicated in a wide range of neurological and psychiatric disorders, yet its functional organization remains incompletely understood. Using direct electrical stimulation combined with the human functional connectome, we mapped the functional architecture of the human thalamus and identified spatially specific stimulation‐evoked responses linked to distinct cortical networks. These findings highlight the central role of thalamo‐cortical circuits in shaping human behavior and provide a framework for developing circuit‐targeted therapeutic strategies.

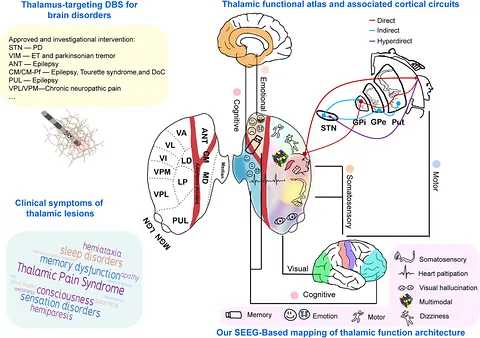

## 1 Introduction

Human thalamus has been recognized as a central hub within thalamo‐cortical loop, subserving complex behaviors. Clinically, dysfunction of thalamo‐cortical circuits is shown to be profoundly implicated in a wide range of neurological and psychiatric diseases, such as Parkinson's disease, Tourette syndrome, epilepsy, major depressive disorder, obsessive‐compulsive disorder [1, 2, 3, 4, 5], and disorders of consciousness [6, 7], which are currently thought of as brain circuit disorders. A number of brain circuit disorders present pharmacological resistance and call for a novel therapy to achieve better clinical benefits. Deep brain stimulation (DBS), by modulating specific brain circuits, has become a promising modality for treating drug‐resistant patients [8, 9, 10]. Considering the central role of the human thalamus in brain function, it is increasingly recognized as an optimal target for DBS in various neurological disorders, including essential tremor [11], epilepsy [12], chronic pain [13], Tourette syndrome [14], and Parkinson's disease [15]. Recent research has also explored thalamic DBS for its safety and efficacy in managing conditions such as traumatic brain injury [16]. Nevertheless, in clinical practice, the outcomes of thalamic DBS vary across patients. Therefore, investigations on the underlying architecture of the thalamo‐cortical network are crucial in neuroscientific research and clinical practice.

The exploration of human thalamic functions originates from the seminal work of Andreas Vesalius centuries ago [17]. From an evolutionary standpoint, the structure and composition of the thalamus shifted from comparatively simple to more complicated and exquisite substrates, adapting to the demands of the environment and the complexities of survival in primates [18]. Traditionally, the primary knowledge of thalamus functions was derived from lesion‐deficit correlation in patients with thalamus lesions, which is rudimentary and lacks precision [19]. Dating back to the 19th century, following the pioneering work of Krause and Cushing, direct electrical stimulation (DES) has been consistently applied as the gold standard for causal mapping of brain functions in vivo [20, 21]. Penfield and Jasper conducted landmark work using DES to investigate human brain function over several decades of neurosurgical practice in the mid‐20th century [22]. A series of animal studies applied DES to investigate thalamus function [23, 24, 25]. DES mapping was largely used to refine the selection and boundaries of cortical regions that are critical for important functions in patients with brain tumors or with intractable epilepsy to avoid neurological deficits [26, 27]. While DES is regarded as the gold standard for investigating human brain function, the thalamus's deep anatomical position makes it challenging to obtain DES data in standard clinical practice, thereby significantly restricting the information gained from thalamic DES. Thus, the exact physiological nature of human thalamus function is still ambiguous.

Stereoelectroencephalography (SEEG), originally designed and developed in the 1960s by J. Talairach and J. Bancaud in France, has evolved into a sophisticated invasive technique for the treatment of drug‐resistant epilepsies [28]. It now offers precise information on the localization of the epileptogenic zone, its relationship with the eloquent cortex, and the feasibility of a customized surgical resection in the presurgical assessment [29]. SEEG provides the advantage of a three‐dimensional, temporally precise analysis of the functional interplay between cortical and subcortical structures, as well as the mechanisms underlying neuromodulation. In particular, SEEG has recently been used to study the thalamo‐cortical loop, providing a deeper understanding the human thalamus and its role in seizures [5, 30]. Building upon our prior studies that highlighted the key role of the anterior thalamic nucleus and subthalamic nucleus during seizure propagation network and optimized the neuromodulation target to guide clinical judgments [31, 32], we recently reported the functional segmentation of basal ganglia in a group of refractory epilepsy patients with at least one SEEG electrode targeting the thalamus and passing through basal ganglia [33]. In the present study, we retrospectively analyzed the clinical responses elicited by DES at various thalamic locations benefiting from temporarily implanted SEEG electrodes into the thalamus during presurgical evaluation. Specifically, the advances of neuroimaging and computational tools have brought the “connectomics revolution” over the past few decades, enabling the explicit study of the thalamus's structure and function from a network perspective [20, 34, 35, 36]. We thereby evaluated whether there is a symptom‐specific intra‐thalamic network by combining functional MRI data derived from the human connectome—resting‐state functional MRI (rs‐fMRI; *n* = 1000). The present study deepens the understanding of the thalamic function architecture and symptom‐specific neural network, promoting potential targeted therapeutic strategies for a wide range of neurological and neuropsychiatric disorders.

## 2 Results

### 2.1 Patient and Electrode Implantation Characteristics

**FIGURE 1:**
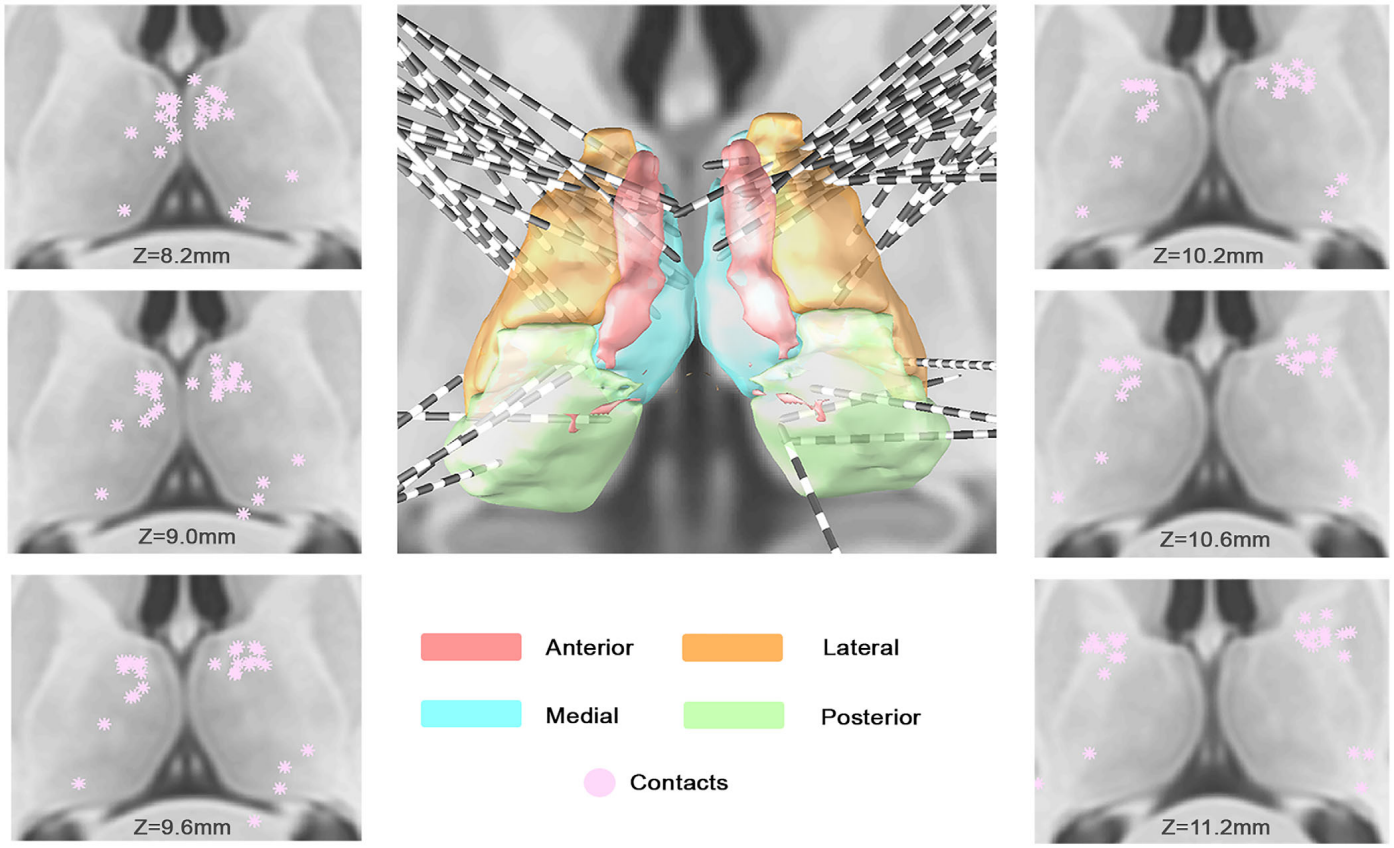
Illustration of grouped SEEG electrode implanted into thalamus in MNI space (ICBM 2009b). 2D and 3D reconstruction of the multisite SEEG electrodes (*n* = 52) passing through the thalamus (red in anterior thalamus, blue in medial thalamus, yellow in lateral thalamus, and green in posterior thalamus) (Table S2). The pink asterisk indicates the location of SEEG contacts. SEEG, stereoelectroencephalography.

Overall, 53 SEEG electrodes from 52 patients were implanted across diverse regions of the thalamus. In total, 138 contacts were retained strictly located within the thalamus for all patients (Table S1). No surgery‐related complications were observed in this patient cohort. The normalized spatial distribution in MNI space of all electrodes within the thalamus is heterogeneous showing more density in the anterior and lateral regions (Figure 1). The precise MNI coordinate of each contact, along with its classification into four well‐accepted thalamic groups—anterior, medial, lateral, or posterior—was used to classify the contact locations (Table S2). Table S1 provides all detailed information on the MNI coordinates of all contacts, including each contact was labeled as corresponding subnuclei using the DISTAL Atlas [37].

### 2.2 The Specificity of Location Eliciting Acute Responses

**FIGURE 2:**
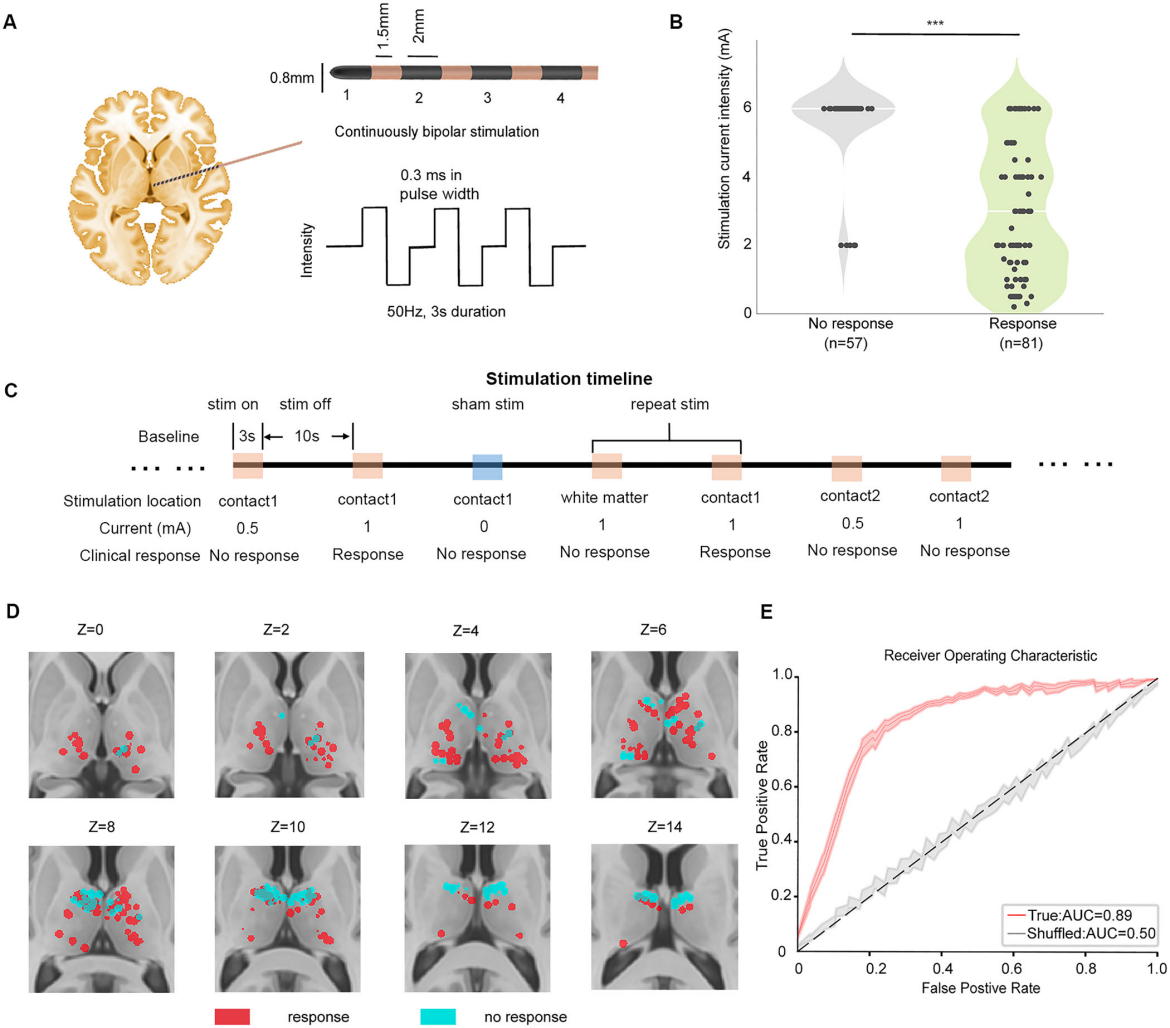
The current intensity and location specificity of the DES. (A) SEEG electrode configuration and stimulation parameters employed in DES. (B) Stimulation current intensity at the sites that elicited non‐active versus active response. (C) Timeline of performing thalamus contacts DES. (D) Spatial distribution of coordinates of contacts with and without clinical responses after DES. (E) ROC curves illustrate the classification of stimulation‐elicited responses versus non‐responses, showing the mean ± SEM over *n* = 1000 randomly sampled features for the true model (red) and the shuffled model (gray). ***p<0.001. DES, direct electrical stimulation; ROC, receiver operating characteristic.

Standard DES procedure (with stimulation parameters of 300 µs, 0.5–6 mA biphasic pulses at 50 Hz, lasting 3 s) was performed for all patients during the chronic SEEG monitoring process (Figure 2A). In total, 81 sites (left = 38, right = 43) reproducibly elicited behavioral or subjective responses and were labeled as active sites, while 57 sites (left = 27, right = 30) did not produce any clinical response and were labeled as non‐active sites. The stimulation current intensity at the sites that elicited active responses was significantly lower than at the nonresponsive sites (mean minimum elicitation threshold [± SD]: 2.93 ± 2.17 mA vs. 6.70 ± 2.15 mA; *t*‐test, *p* < 0.001) (Figure 2B).

All the included response in this study is validated with sham stimulations and repeated stimulations procedures during stimulation procedures (Figure 2C). Active and non‐active sites were equally distributed across the two hemispheres (*χ* ^2^ *p* = 0.97) (Figure 2D). Moreover, to further evaluate the specificity of sites within the thalamus eliciting acute responses (active vs. non‐active) by DES, we selected the spatial position (x, y, and z coordinate in MNI space) and corresponding stimulation intensity as features for all 81 contact sites. By leveraging two cross‐validated supervised machine learning models that were trained on 80% of the data and tested on the remaining 20%, and by randomly permuting class labels 1000 times, we found that active sites could be well distinguished from non‐active sites (accuracy: mean = 0.82, SD = 0.04; area under the curve [AUC]: mean = 0.89, SD = 0.03; Figure 2E), supporting location‐specific responses.

### 2.3 Acute Clinical Responses Elicited by Thalamus DES

**FIGURE 3:**
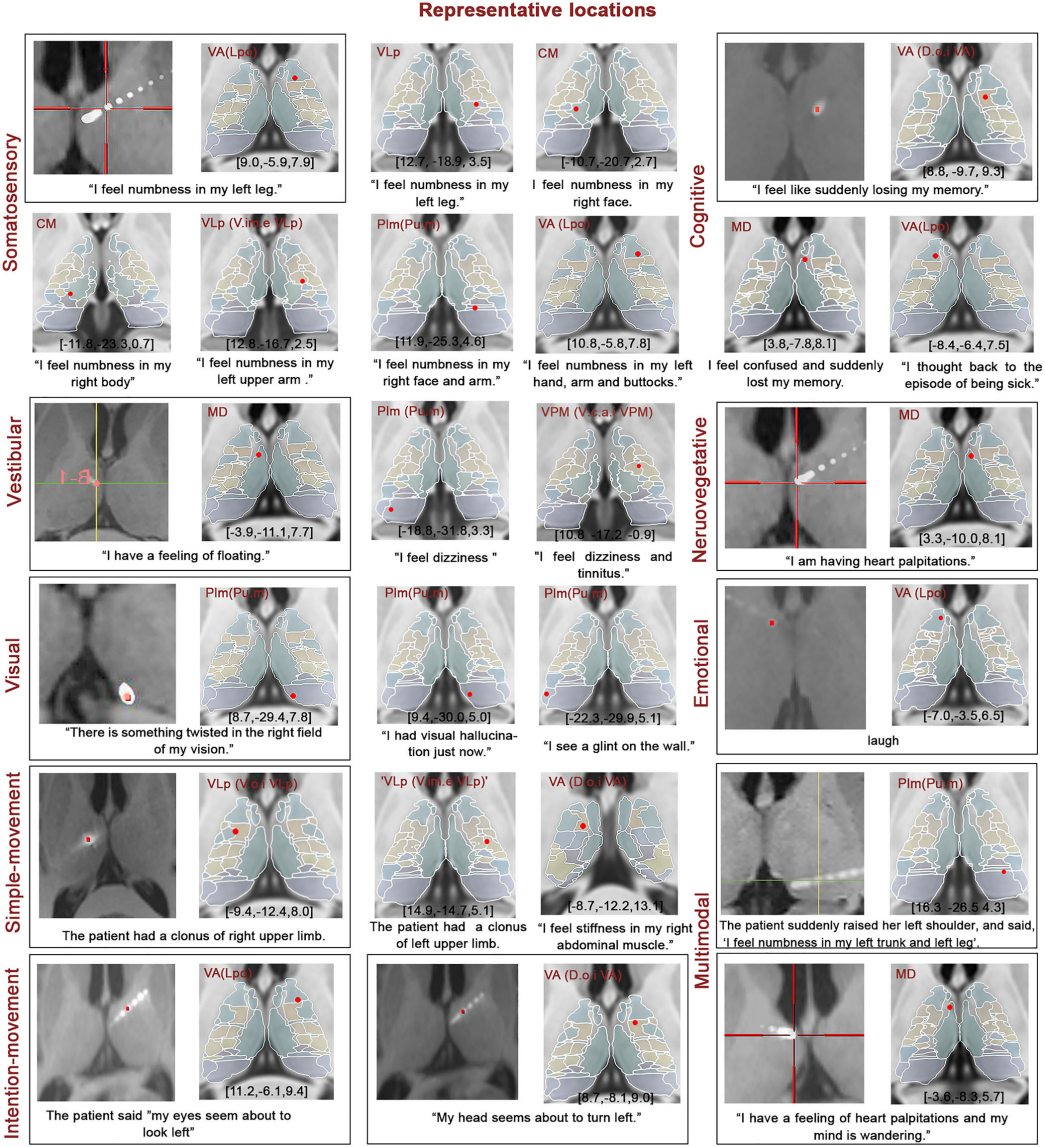
Representative acute responses arising from DES of the thalamus. These panels show examples of individual contact sites in various regions within the thalamus in MNI space, along with detailed reports and/or objective responses from patients during stimulation of the corresponding sites (red dots). The MNI coordinate of each location was shown in combination with thalamic nuclei. CM, central median nucleus; MD, mediodorsal nucleus; Plm (Pu.m), medial pulvinar nucleus (pulvinar mediale); VA (D.o.i.VA), ventral anterior nucleus dorso‐oralis internus; VA (Lpo), ventral anterior nucleus (lateropolaris); VLp (V.im.e VLp), ventral‐intermedius externus ventral lateral posterior nucleus; VPM, ventral medial nucleus.

Intriguingly, DES of 81 distributed contacts within thalamus evoked a broad spectrum of in‐situ objective and subjective symptom responses spanning fundamental functions such as sensory and motor processing, extending to complex neural operations encompassing neurovegetative regulation, cognitive processing, emotional modulation, and multimodal responsiveness. The original reports were documented (Table S1). Representative responses are illustrated in Figure 3.

**FIGURE 4:**
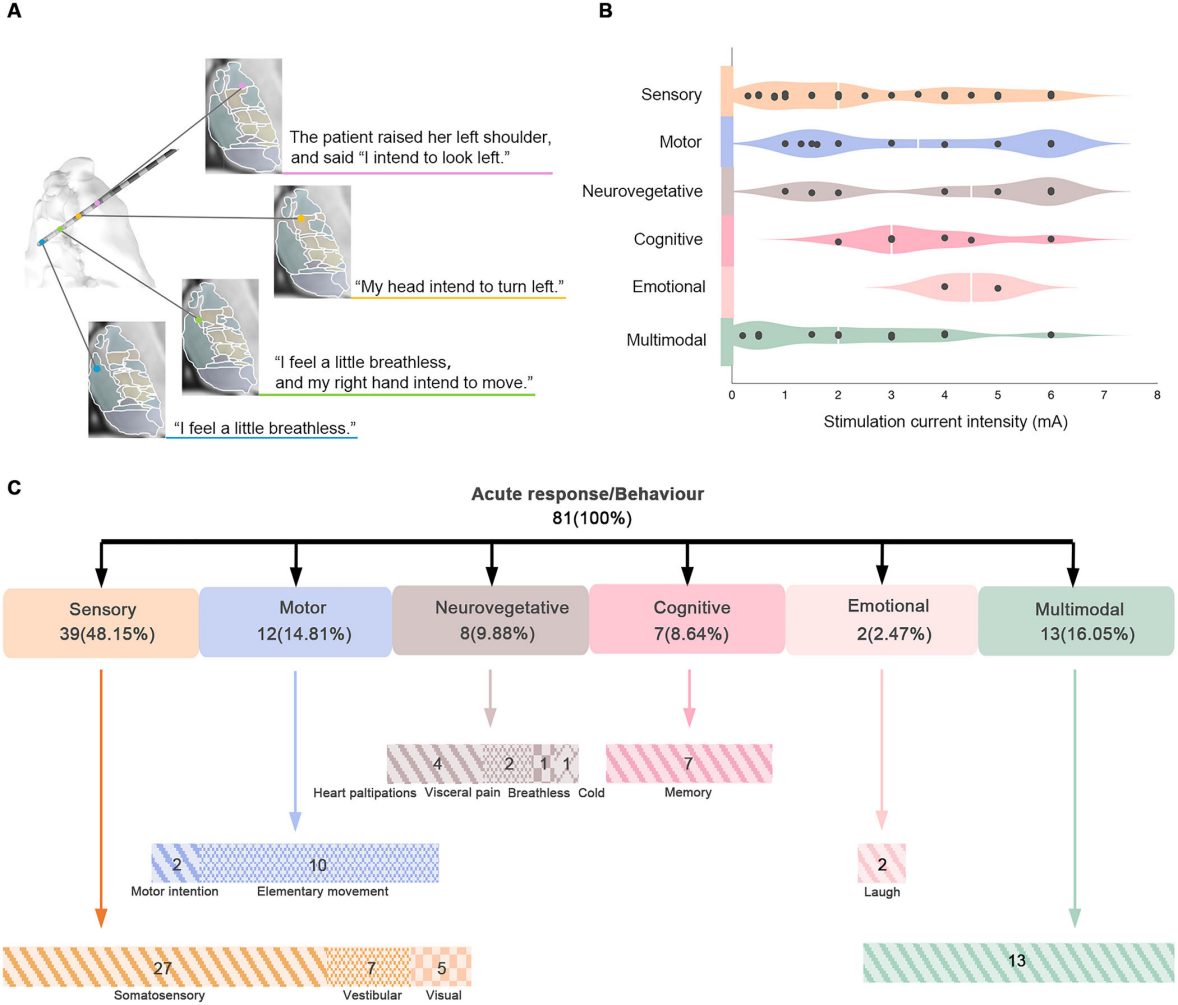
Categories of acute clinical responses elicited across distributed contacts. (A) Example of different clinical responses elicited by stimulating adjacent thalamic contacts on same SEEG electrode. (B) Stimulation current intensity elicited responses in each category and spatial distribution of repertoire responses. (C) Number of elicited acute responses/behaviors classified into six categories.

We saw different responses while stimulating adjacent contacts within the thalamus, which preliminarily demonstrated the spatial specificity of thalamus function (Figure 4A). Of the 81 responsive stimulation contacts, 68 evoked a single response while 13 exhibited multiple responses. The clinical responses were further classified into the following semiological categories [33, 38, 39, 40]: (i) sensory responses (including somatosensory, vestibular, and visual sensation); (ii) motor symptoms (including simple motor behaviors, intentional and eye movements); (iii) neurovegetative symptoms (including heart palpitations, visceral pain, and breathless); (iv) cognitive response (including mind blank and time perception); (v) emotional responses (including laugh, anxiety, and grief); and (vi) multimodal responses (symptoms belonging to two or more than two of the above categories). The stimulation current intensity was 2.68 ± 1.87 mA (sensory responses), 3.62 ± 2.10 mA (motor responses), 3.93 ± 2.15 mA (neurovegetative responses), 3.64 ± 1.31 mA (cognitive responses), 4.50 ± 0.71 mA (emotional responses), and 2.13 ± 1.79 mA (multimodal responses), as shown in Figure 4B (ANOVA, *F* = 1.861, *p* > 0.05).

Sensory responses were observed in 20 patients at 39 sites (Figure 4C). The elicited somatosensory responses were described as numbness and pain covering the contralateral face, head, limb, and body (27 sites) in the lateral group of thalamic stimulation. At the nucleus level, these responses were distributed across multiple thalamic nuclei, predominantly involving ventral tier nuclei classically associated with somatosensory processing, including ventral posterior and ventral lateral regions. In addition, somatosensory responses were also observed in ventral anterior nucleus (VA), intralaminar nuclei (e.g., the centromedian nucleus, CM), and posterior thalamic regions including the pulvinar. Vestibular sensory symptoms were characterized by feelings of floating and dizziness, with all stimulation sites situated within the lateral group (7 sites), and were most frequently associated with ventral posterior and ventral lateral nuclei, with additional involvement of mediodorsal nucleus (MD), VA, and posterior thalamic nuclei including the pulvinar. Visual perceptions were detected at five sites and described as “There is something twisted in the right field of my vision,” “I was having a visual hallucination just now,” and “I see a glint on the wall” during stimulation of the medial and lateral pulvinar nucleus.

Motor symptoms were observed in six patients at 12 different sites (Figure 4C), constituting the second most frequent response. Simple motor behaviors, such as elevation of the contralateral upper limb, stiffness, and clonus, were triggered in six locations, and at the nucleus level, were predominantly associated with ventral tier thalamic nuclei, particularly the ventral lateral and VA, with additional involvement of ventral posterior nuclei. Motor intention was detected in two sites in the lateral group of the thalamus, with the patient describing their experience as, “my head seems about to turn left,” when the contralateral side was stimulated. At the nucleus level, motor intention‐related responses were localized to VA. In addition, eye movements were observed. One patient turned bilateral eye movement to the left when the right side of the lateral group of the thalamus stimulated while another patient presented with limited eye mobility in both eyes upon stimulation of the posterior group of the thalamus. At the nucleus level, eye movement‐related responses predominantly involved VA, with additional localization to ventral posterior nuclei and posterior thalamic regions including the pulvinar.

Neurovegetative responses were observed in six patients at eight different sites (Figure 4C). Three patients reported experiencing heart palpitations, one patient reported having dyspnea with a sensation of breathlessness, one patient experienced “feeling cold,” and one patient reported “abdominal burning pain” when stimulated the medial group and lateral group of the thalamus. At the nucleus level, neurovegetative responses were primarily associated with stimulation of the CM and ventral posterior lateral nucleus, with additional involvement of MD and VA.

Cognitive responses, including time perception and mind blank, were observed in four patients at seven different sites (Figure 4C). Episodic mind blank was obtained with the following descriptions: “I feel like I'm in a strange place, like a station, and I fell into an empty and restless state,” “Many scenes flashed through my mind, and there seemed to be a white rectangular object in the scene,” “I feel like suddenly mind blank,” and “I thought back to the episode of being sick.” At the nucleus level, the three sites were located in MD and VA, respectively.

Emotional responses, including laughter, were observed in one patient while stimulating two different sites within VA (Figure 4C).

Amidst these observations, we identified instances of multimodal reactions, characterized by the simultaneous activation of two or more categories, in eight patients at 13 different sites (Figure 4C). These sites exhibited a spatial distribution within distinct groups of thalamic nuclei. In five sites among three patients, both motor and sensory responses were observed. As patients elevated their upper bodies, they articulated, “I feel numbness in my left trunk and left leg” when stimulated in the ventral posterior lateral nucleus and medial pulvinar nucleus. Notably, during VA stimulation, individuals reported a combination of numbness and heart palpitations. One patient experienced breathlessness, and their right hand appeared poised for movement, accompanied by heart palpitations during VA stimulation, another patient reported a sensation of heart palpitations and mind wandering upon MD stimulation. In another case, a patient reported heart palpitations coupled with feelings of sadness and upset in response to pulvinar stimulation. At the nucleus level, multimodal responses were predominantly associated with stimulation of the MD and VA, as well as posterior thalamic regions including the pulvinar, with additional involvement of ventral posterior nuclei.

### 2.4 Response‐Specific Probabilistic Maps

**FIGURE 5:**
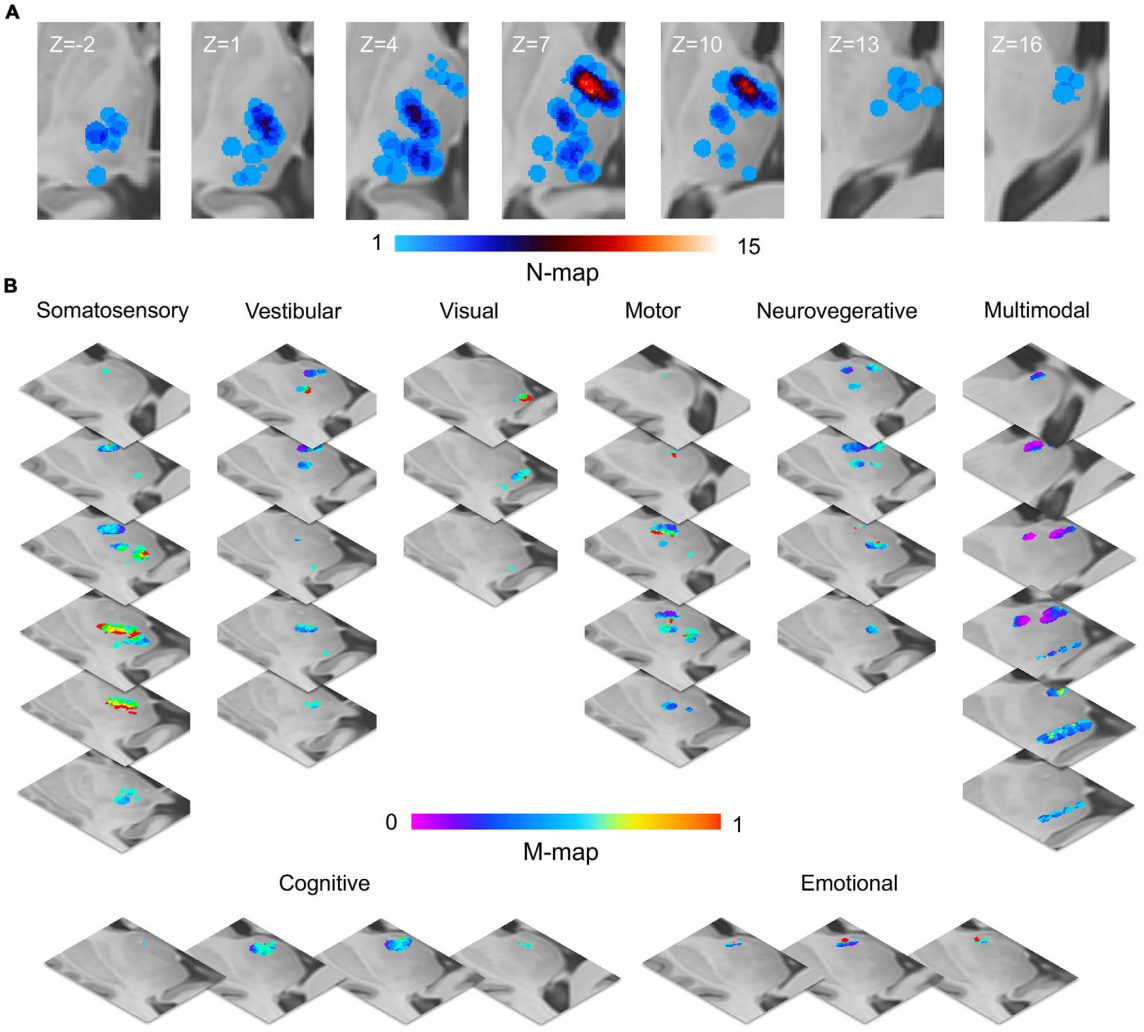
N‐map and M‐map on thalamus stimulation in MNI space. (A) N‐map, incorporating ROIs of all 81 contacts, warm colors indicate that the voxel was stimulated more frequently. (B) M‐map, summed informed ROIs of specific symptoms, warm colors suggest that the voxel's stimulation is more closely associated with the corresponding symptom. M‐map, Mean‐Image; ROI, region of interest.

To quantitatively test the symptom‐specific functional topography of thalamus, we created the N‐map and Mean‐Image (M‐map). The N‐map represents the number of occurrences of voxel stimulation by overlapping patient‐specific region of interest (ROI) generated as a MNI coordinate‐based 3 mm radius sphere (Figure 5A). The M‐map, calculated by averaging the sum of clinical responses at each voxel—this process yielded a raw average frequency map, reflecting probabilistic maps of specific responses at each voxel level (Figure 5B).

The M‐map revealed distinct spatial distribution patterns across response categories. Vestibular, visual, motor, cognitive, and emotional responses showed relatively focal probability peaks within specific thalamic regions, whereas somatosensory, neurovegetative, and multimodal responses exhibited more spatially distributed probability peaks, indicating a lower degree of anatomical specificity (Figure 5B). The somatosensory had its peak value in the medial and lateral thalamus, the vestibular had its peak value in the lateral thalamus, the visual had its peak in posterior thalamus, the motor had its peak value in medial and lateral thalamus, the neurovegetative had its peak value in the medial thalamus, the cognitive and emotional had its peak value in the anterior thalamus, and the multimodal had its peak value in the medial and posterior thalamus. These results primary revealed the intrinsic functional organization of the human thalamus.

### 2.5 Symptom‐Specific Intra‐Thalamic Network

**FIGURE 6:**
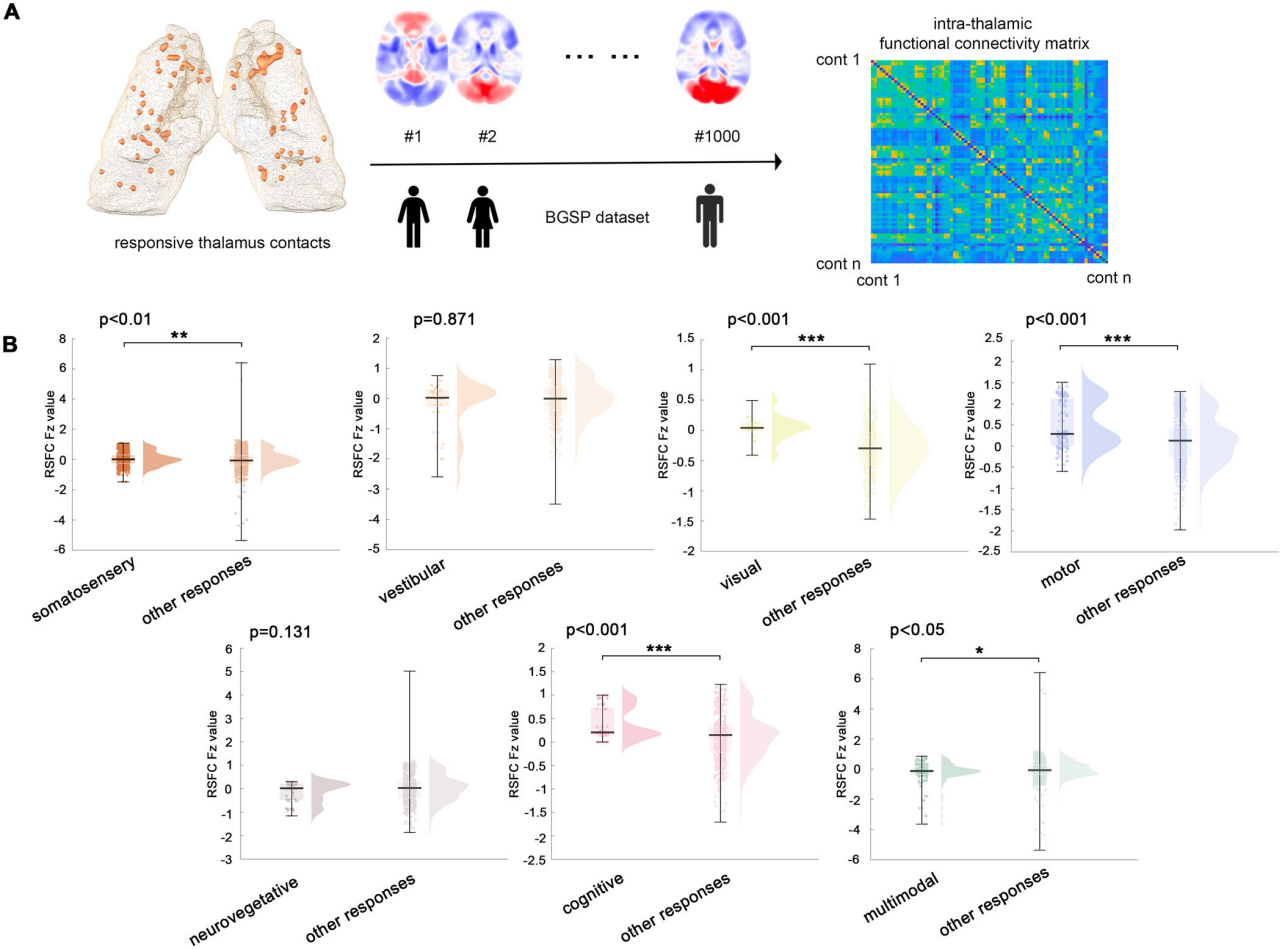
Intra‐thalamic symptom‐specific functional network demonstrated by contacts elicited similar responses. (A) Protocol to estimate the functional connectivity between the contacts within the thalamus that elicited various clinical responses. The colored spheres within the thalamus represent contacts associated with different clinical responses. Matrix on the right indicates the functional connectivity strength of the responsive contacts. (B) Raincloud plot of the strength of functional connection between contacts elicited similar clinical responses and different clinical responses. Statistical analysis revealed a significant discrepancy in functional connectivity between contacts that elicited similar responses and those that elicited different clinical responses across various symptoms. Emotion responses were not included due to the limited amount of data (*n* = 2). *p < 0.05, **p < 0.01, ***p < 0.001.

Moreover, we assessed the functional connectivity between all the contacts that elicited clinical responses to determine whether there is an intra‐thalamic intrinsic functional network associated with each specific clinical response (Figure 6A). Also, using the active sites for each category response, we estimated the symptom‐specific large‐scale whole‐brain functional network derived from human connectome data (*n* = 1000).

After eliminating the influence of the distance between contacts that elicited clinical responses, we observed that contacts eliciting similar responses were more closely connected than those that elicited different response. This pattern was evident in several stimulation‐elicited responses, including somatosensory, motor, visual, cognitive, and multimodal responses. However, contacts that elicited vestibular and neurovegetative responses did not show a significant closer connection, likely due to the limited sample size (Figure 6B). The closer connection between thalamus contacts that elicited similar responses potentially suggests a symptom‐specific intrinsic network within the thalamus.

## 3 Discussion

This study represents the inaugural and presently the most exhaustive analysis of the functional architecture of the human thalamus via multisite DES using SEEG in combination with human connectome data. From the classical localizationist model to the connectionist and associationist view, a cerebral‐centric perspective has long dominated the understanding of brain functions' origins [41]. Our results indicate that the thalamus also harbors functional capabilities. This data‐driven exploration of clinical responses stands as the original and authentic portrayal of physiologically evoked clinical responses not solely the anticipated spectrum of primarily sensory, motor, and neurovegetative responses, but also high‐order functions including cognitive perceptions, affective manifestations, and psychopathological manifestations. Our data provide the direct substantiation for the complex functional architecture of human thalamus and advancing current understanding of its role.

**FIGURE 7:**
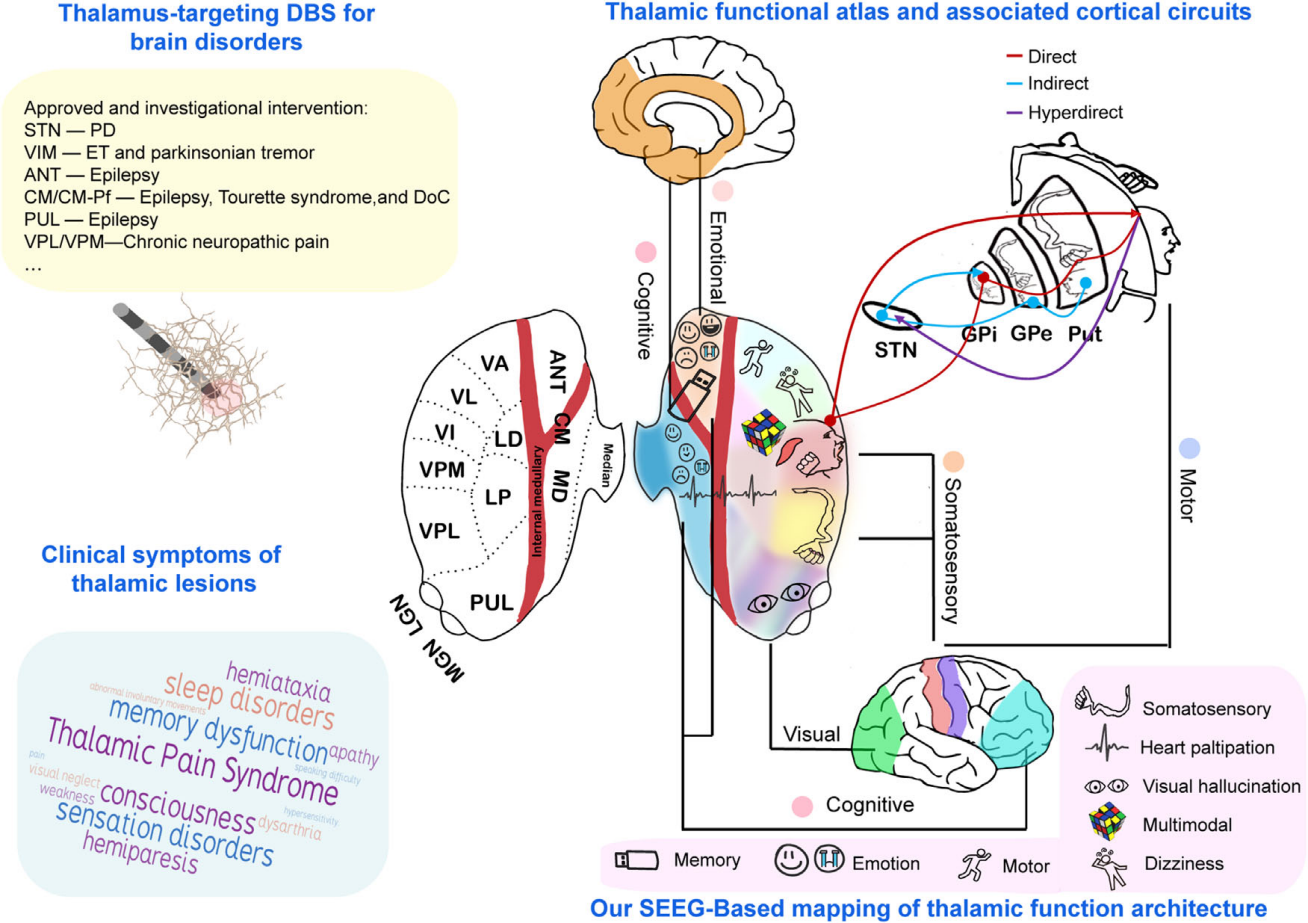
A schematic map of the functional architecture of the human thalamus. Current canonical understanding of cortico‐thalamic circuits, the syndromes associated with thalamic lesions, and thalamic stimulation for a range of neuropsychiatric disorders. ANT, anterior nucleus of the thalamus; CM‐Pf, centromedianparafascicular complex; DBS, deep brain stimulation; DoC, disorders of consciousness; ET, essential tremor; GPe, external globus pallidus; GPi, internal globus pallidus; LD, lateral dorsal nucleus; LGN, lateral geniculate nucleus; LP, lateral posterior nucleus; MGN, medial geniculate nucleus; PD, Parkinson's disease; PUL, pulvinar nucleus; Put, putamen; SEEG, stereoelectroencephalography; STN, subthalamic nucleus; VI/VIM, ventral intermediate nucleus; VL, ventral lateral nucleus; VPL, ventral posterolateral nucleus.

By integrating the present DES findings with evidence from prior anatomical, clinical, and neuromodulation studies, we propose a schematic model summarizing the functional architecture of the human thalamus (Figure 7). This model highlights four complementary dimensions of thalamic organization: (i) well‐established cortico‐subcortical loops, (ii) symptom‐specific functional connectivity topography identified by stimulation‐evoked responses, (iii) clinical syndromes associated with focal thalamic lesions, and (iv) established thalamic DBS targets for neurological and neuropsychiatric disorders. Rather than suggesting a one‐to‐one mapping between individual thalamic nuclei and isolated functions, this framework emphasizes that human thalamic function is organized through both spatially specific thalamic territories and distributed network‐level connectivity.

When viewed within classical, region‐level frameworks, several of our observations align with established knowledge. Visual responses were preferentially localized to posterior thalamic regions, in line with the established role of posterior thalamus in visual processing [42, 43]. Cognitive and emotional responses were largely confined to the anterior thalamus, consistent with classical views linking anterior thalamic territories to limbic and higher‐order cognitive functions [44, 45, 46]. Vestibular responses exhibited a focal probability peak in the lateral thalamus but were spatially distributed across lateral and medial regions, supporting the notion that vestibular processing within the thalamus involves broader thalamic territories rather than a single discrete nucleus [47, 48]. Somatosensory responses were widely distributed across the thalamus, consistent with classical descriptions of overlapping thalamic representations rather than a focal relay [49, 50].

Beyond these consistencies, several response categories extended classical thalamic models by revealing cross‐territorial organization. Motor responses showed probability peaks across medial and lateral thalamic regions, with limited extension into the anterior thalamus, suggesting that motor‐related processing engages multiple thalamic territories [51]. Neurovegetative and multimodal responses likewise displayed distributed patterns spanning medial and posterior thalamic regions, highlighting an integrative role of the thalamus across functional domains [52, 53, 54].

The somatotopic organization of primary motor and somatosensory cortices is a widely recognized and commonly acknowledged rule. Despite that prior studies on somatotopic organization mostly focus on the cortex [55], anatomical and electrophysiological investigations have revealed that subcortical structures, such as the striatum, subthalamic nucleus, external and internal segments of the globus pallidus, exhibit distinct homunculus‐like somatotopic organization [55, 56]. The current study suggests that the thalamus also harbors a homunculus topographical arrangement, and a comprehensive delineation of this intrinsic topography necessitates further investigations for complete elucidation.

Our study showed that stimulation primarily induced sensory‐related responses, prominently observed in the lateral group of the thalamus but also CM nucleus in the middle of thalamus. The somatosensory responses evoked were mostly characterized by numbness, aligning coherently with the prevailing sensory deficits of numbness observed in patients with thalamic stroke [57, 58]. Of note, the visual response, which was induced in three distinct sites loci within the pulvinar, owing to the well‐established central role in visual processing [59, 60, 61]. The third distinctive response is the vestibular sensory response, which is consistent with a previous study illustrating how thalamic subnuclei process a distinctive verticality about direction [62, 63, 64]. Also, our previous study showed that the thalamus serves as a unique relay station for vestibular input to the cortex [65]. Beyond its well‐established sensory roles, our motor‐related findings in both the lateral thalamus (particularly the VA) and the medial thalamus (particularly the MD) support the classical functional attribution of the motor thalamus, while suggesting a broader, cross‐territorial organization beyond a strictly localized relay. According to our data, thalamic stimulation triggered not only simple motor responses but intricate movements, purposeful actions, and ocular motions. Insights gleaned from animal studies posit the engagement of thalamic internal lamina cells in visuomotor integration [66, 67]. Moreover, substantial and high‐quality evidence supports the use of thalamic ventral intermediate nucleus stimulation to alleviate tremors [68], and motor thalamus DES to improve motor control [69].

So far, limited literature exists regarding thalamic injuries manifesting neurovegetative symptom and presenting as ventilatory disorders or arrhythmia [70, 71]. The MD is widely acknowledged to signify sympathetic reactivity, potentially accounting for the observed instances of induced dyspnea and tachycardia [72, 73]. The centromedian‐parafascicular nuclei complex (CM‐Pf) is known to play a role in restoring consciousness, and studies have shown that CM‐Pf stimulation can promote the recovery of independent functionality in patients with minimally conscious states [74]. Our observation of reproducible neurovegetative responses elicited from MD and CM stimulation contributes to elucidating the potential role of the thalamus in neurovegetative symptom across a spectrum of diseases.

Specifically, the observation of cognitive responses mainly located in VA and MD in the current study supports the thalamus as the essential partner of the cognitive network [75]. Indeed, human lesion studies strongly suggest that the thalamus is involved in a wide range of cognitive functions, which suggests the role of VA and MD in influencing complex cognitive functions and indicates the potential promising therapeutic targets for ameliorating cognitive deficits in conditions encompassing neuropsychiatric and neurodegenerative disorders [76]. Our research revealed that thalamic stimulation can elicit positive emotional responses such as laughter, as well as negative emotions linked to sadness and grief. Previous research delves into the thalamic‐amygdala circuit's role in extinguishing remote fear memories [25, 77]. We identified thalamus as a critical region that plays an important role in affective state control. In parallel, psychic responses were induced by sites spatially separately from thalamic nuclei. Intriguingly, previous studies report hyperconnectivity between the thalamus and sensorimotor cortices in psychedelic states, correlating with altered visual and auditory perceptions [78], which might offer an explanation for the phenomena we observed.

In the present study, we found multimodal response predominantly in the MD and medial pulvinar nucleus. Beyond conventional point of view that each thalamic nucleus is dedicated to a specific function, mounting studies evidencing the thalamic hub nuclei serve multiple functions. Several thalamic nuclei, including the anterior, MD, intralaminar, ventrolateral, ventroposterior, and pulvinar nuclei, have demonstrated robust connector hub characteristics, even stronger than cortical connector hubs [79], which further implies that these thalamic nuclei occupy a privileged position to support diverse functions across multiple functional networks [79, 80]. Previous tracing studies have shown that the medial pulvinar nucleus exhibits reciprocal connectivity with the inferior parietal lobule, inferior temporal cortex, frontal eye fields, and the visual attention network [81]. In addition, the MD nucleus has been noted for its extensive functional connectivity with numerous brain regions, encompassing the frontal cortex, the cingulate cortex, and the insular cortex [82, 83]. Roy et al. [34] have proposed the concept of “subnetwork” within thalamus nuclei. These “subnetwork” consists of groups of cells that exhibit specific patterns and serve distinct functions. Our findings suggest that hub nuclei may harbor a greater number of “subnetworks,” which further enable them to participate in multiple functions across various domains.

Compared to the well‐known trans‐thalamic network connecting cortical areas, the intra‐thalamic network has been relatively overlooked [84]. Given the role of the thalamus itself has been explored in a number of studies [85], we should take a closer look at the intra‐thalamic mechanisms of the neurological symptoms. The present study identified a closer connection between thalamic contacts that elicited similar somatosensory, visual, motor, cognitive, and multimodal symptoms. This finding suggests that the intra‐thalamic network may have impact in a wide range of clinical symptoms. Future studies should further explore these networks to better understand their significance and underlying mechanisms in human brain functions.

The study's constraints warrant careful consideration. First, it is essential to acknowledge the matter of inadequate thalamic coverage as it is inherently associated with limited sampling. Second, the relatively brief stimulation durations employed in this investigation (3 s) could potentially prove inadequate for eliciting the complete spectrum of high‐order responses. Third, with the size limitation of the 2 mm electrode across the nucleus, there is a challenge that the elicited responses may tend to extend beyond individual subnucleus boundaries. Although stimulation sites were anatomically localized at the nucleus and subnucleus level, stimulation‐induced responses frequently spanned multiple adjacent nuclei. Given the small volume and close spatial proximity of thalamic nuclei, as well as the volume of tissue activated by DES, individual nuclei could not be treated as independent functional units for primary statistical analyses. Finally, the DES data in this study were derived from patients with refractory epilepsy, in whom chronic disease may have reshaped thalamic networks. Although prior research suggests that, with rigorous controls, epilepsy‐affected brains can serve as reasonable proxies for normal brain function [86], these disease‐related adaptations still underscore the need for caution when generalizing findings beyond the epileptic brain.

In summary, our observation reveals that the functional architecture of thalamus closely mirrors that of the entire cortex. This underscores the foundational role of thalamo‐cortical circuits in shaping human behavior. Furthermore, this identification of symptom‐specific locations within the thalamus and corresponding circuits holds the potential for refining the current treatment targets, finding novel targets, and ultimately designing a symptom‐specific neuromodulation strategy for neuropsychiatric disorders.

## 4 Materials and Methods

### 4.1 Subjects

For this study, we retrospectively reviewed the stimulation data from refractory focal epilepsy patients who underwent SEEG between 2017 and 2023 at Xuanwu Hospital, Capital Medical University, Beijing, China. All the patients required continuous SEEG recordings to precisely localize the epileptogenic zone or map the eloquent cortex because of insufficient information from comprehensive noninvasive evaluations, including detailed clinical history, seizure semiology, video scalp electroencephalogram, MRI and/or positron emission tomography. The number and placement of SEEG electrodes were determined based on clinical needs, with electrodes implanted into the thalamus following subtle angle adjustments. Thalamic trajectories were specifically designed to assess functional connectivity between cortical epileptogenic zones and thalamus while maintaining strict safety protocols.

All the patients who met the following criteria were included in the final analysis: (i) At least one SEEG electrode was targeting the thalamus to map the cortico‐subcortical epileptic network or refine the stimulation target. (ii) DES was performed on the electrode targeting the thalamus, and the video‐SEEG recordings and subjective or objective responses evoked were documented in detail. (iii) There was no significant brain deformation due to lesions or encephalomalacia.

### 4.2 Standard Protocol Approvals, Registrations, and Patient Consents

All procedures performed in studies involving human participants were in accordance with the ethical standards of the Ethics Committee of Xuanwu Hospital, Capital Medical University ([2024]126) and with the 1964 Helsinki declaration and its later amendments or comparable ethical standards. The study derives from our prior series of works [33, 87, 88], also leveraged an ongoing in‐hospital clinical trial (ChiCTR2100047743) involving the implantation of intracranial electrodes in DRE.

Each patient representative voluntarily provided written informed consent to participate in this study. To ensure the confidentiality of the participants’ information, all data were deidentified, and arbitrary codes were assigned to each patient's data.

### 4.3 SEEG Implantation and Reconstruction

All patients underwent high‐resolution T1‐weighted MRI scans (3.0 T, Siemens) before surgery and three‐dimensional CT scans within 24 h after SEEG implantation surgery to verify electrode placement. Under general anesthesia, the implantation of SEEG electrodes was performed using an oblique approach. The detailed implantation procedure for SEEG electrodes has been described in our previous publications [32, 87, 89]. The multisite SEEG electrodes were semirigid platinum/iridium, and they were 0.8 mm in diameter with 8–16 contacts, and 2 mm in length, with 1.5 mm apart (Sinovation, China).

For each patient, we calculated the locations of these contacts using the Lead‐DBS software (https://www.lead‐dbs.org/). First, we co‐registered the postoperative CT scan to preoperative T1‐weighted image using two‐stage linear registrations implemented in Advanced Normalization Tools (http://stnava.github.io/ANTs/). Second, we spatially normalized these scans to MNI space (MNI152 ICBM2019b) based on the preoperative T1‐weighted image using Advanced Normalization Tools. Third, we corrected these reconstructions by using the “brain shift correction” module in Lead‐DBS. Fourth, we reconstructed the positions and trajectories of the SEEG electrodes based on postoperative CT scan.

### 4.4 The Electrode Localization Within Thalamus

To reveal the anatomo‐functional correlation of contacts within thalamus, two approaches were employed. The original coordinates of all electrode contacts from each included patient serve as the structural foundation for functional mapping. Specifically, the coordinate of each contact inside thalamus was provided.

More specifically, we meticulously reviewed all the contacts’ locations by comparing the normalized CT and MRI in MNI space to co‐registered images in individual spaces. We visually confirmed the positions of the contacts within the thalamus. To avoid the potential effect of exciting adjacent fibers, we carefully performed visual inspections. Any contact located in the internal capsule was excluded for further analysis.

Next, the anatomical correlation between each contact site with the known thalamic subnuclei was assessed using the DISTAL Atlas [37]. The DISTAL Atlas is the most common high‐resolution template of subcortical structures in the MNI space used as a reference for the manual localization and segmentation of targets based on histology, structural imaging, and diffusion‐weighted imaging [90]. According to the DISTAL Atlas, each contact was labeled as corresponding subnuclei. We created masks for each subnuclei and used them to define the spatial relationship of each contact within the thalamus along the SEEG trajectories. In addition, the thalamus was divided into four subgroups. Given the limited volume of most thalamic subnuclei, we used these four subgroups including anterior, lateral, medial, and posterior parts with varied numbers of subnuclei in combination with specific subnuclei in subsequent analyses.

### 4.5 SEEG Recordings and Stimulation Procedure

Routinely, SEEG signals were recorded for 7–14 days via a Micromed (*n* = 27) sampling data at 1024 Hz or Neuracle (*n* = 25) sampling data at 1000 Hz video‐EEG monitoring system to capture at least three habitual clinical seizures after surgery. During chronic SEEG monitoring, the DES procedure was conducted as a routine part of the standard presurgical assessment, as described in our previous study [33, 40]. Electrical pulses were generated with Grass S88 (SUI‐7, Astro‐Med Inc.). Given the SEEG contact surface and volume (Sinovation electrodes, approximately 0.05 cm^2^), the charge density was between 3 and 36 µC/cm^2^, all parameters were carefully adjusted to prevent any tissue damage, prioritizing safety and effectiveness [91, 92].

Typical bipolar high‐frequency stimulations (frequency: 50 Hz; pulse width: 0.3 ms; duration: 3 s) of two serial and adjacent contacts were carried out using charge‐balanced biphasic rectangular stimuli of alternating polarity. Real‐time electrophysiological data monitoring was performed concurrently. The current intensity initially ranged from 0.5 to 1.0 mA and was increased by 0.5 mA up to a maximum of 6 mA, or when electrical after‐discharges or behavioral responses were observed.

During stimulation, the patients were asked to sit in bed and remain in a resting state. They were blinded to the stimulation location, parameters, and the start and end time points of the stimulation. Patients were asked to report any elicited experiences or subjective responses/behaviors.

### 4.6 Internal Controls of DES to Recognize Evoked Responses

Two approaches were utilized to ensure the reliability of responses elicited by the stimulation: (i) sham stimulation and (ii) repeated stimulations. If the patient reported any clinical response elicited by stimulation, a sham stimulation was then performed on the same contact to verify the response. Only responses elicited by veridical stimulation, but absent during sham stimulation, were considered reliable in this study.

When subjective or objective responses were observed during stimulation of a contact within the thalamus, the same stimulation parameters were applied to a contact within the white matter on a different electrode. Repeated stimulation of the thalamic contact using the same parameters was subsequently performed. A clinical response was considered dependable only if it was consistently reproducible on the thalamic contact and absent during stimulation of the white matter contacts.

Notably, trials with after‐discharges were excluded for further analysis. In addition, the trials that showed cortical synchronic activity were also excluded by visual inspection to ensure a focused reflection of intrinsic in‐situ thalamic function. All responses induced by stimulation were video recorded and stored in clinical report documents with the patient's descriptions of their experiences (Table S1).

### 4.7 Evaluation of the Specificity of Contact Sites Eliciting Acute Responses

To test the reliability of the response versus no response, a classifier was deployed based on each contact position (x, y, and z coordinate in MNI space) and stimulation intensity. In brief, all the electrode contacts were labeled as active or non‐active responses according to the observation from stimulation procedure. Spatial location coordinates and stimulation parameters were used as features to evaluate model performance with respect to stimulation location and its association with clinical responses. The modeling pipeline includes classification using penalized logistic regression, with the model trained on 80% of the data and tested on the remaining 20%. To validate consistent AUC scores and feature importance, this training/testing cycle was repeated 1000 times.

### 4.8 Evaluation of Response‐Specific Probabilistic Frequency

For each site eliciting specific acute symptom within the thalamus, an ROI was generated as a MNI coordinate‐based radius sphere. We selected a distance of 3 mm radius sphere to ensure sufficient coverage around the contact without including irrelevant space, following a common approach used in other studies [32, 93]. These personalized ROIs within the thalamus were designated as thalamus seeds.

To describe how often a certain voxel was stimulated in a set of stimulation volumes, we created an N‐map [94]. For binary personalized ROIs, the N‐map is generated by adding all ROIs up, allowing for the value of each voxel of the N‐map to reflect the number of times it was covered by ROIs from the dataset. Further, to reflect whether specific response can be elicited upon stimulating single voxel, we generated the M‐map [95]. We define voxels in ROIs which can elicit specific response 1 and the rest 0 thus made the ROIs into informed ROIs. These informed ROIs were subsequently summed and a summation image in which each voxel represents the sum of all responses caused by the ROIs which stimulated this voxel. Later, each voxel of this summation image is divided by how often they were stimulated, and the M‐map represents the average outcome caused by stimulation for each voxel. We intentionally removed voxels with *N* value at 1 to exclude the biased peak value of the M‐map could be generated by a single voxel with specific symptom.

### 4.9 Estimation of Functional Specific Thalamo‐Cortical Network

In this study, we utilized a large normative rs‐fMRI dataset to evaluate the strength of connectivity between contacts that elicited clinical responses and to identify the specific brain areas to which these contacts are connected.

The human connectome (rs‐fMRI, *n* = 1000) dataset applied in this study is obtained from the Brain Genomics Superstruct Project (BGSP) (https://dataverse.harvard.edu/dataverse/GSP) [96, 97]. The application of normative connectomes is instrumental in associating various specific neurological or psychiatric symptoms with networks [98]. The connectome data have been preprocessed by Al‐Fatly et al. [99] and are embedded in the Lead Connectome Mapper (https://netstim.gitbook.io/leaddbs/connectomics/lead‐mapper). In brief, the preprocessing approach involves motion correction (SPM‐based), detrending, regression of white matter and cerebrospinal fluid signals, as well as motion parameters, and bandpass filtering (0.009–0.08 Hz) of time series [100, 101]. The voxel size resolution was set at 2 mm, and spatial smoothing was implemented using a Gaussian kernel with an 8 mm full width at half maximum.

We calculated the functional connectivity matrix to evaluate the strength of connectivity between responsive contacts. Each of the responsive contacts is considered as a seed, and the correlation matrix between the seeds is generated by calculating the Pearson's correlation coefficients of the averaged time series of all voxels within each seed. The result was then Fisher r‐to‐z transformed.

### 4.10 Statistics

To examine the symptom‐specific inter‐thalamic network associated with specific clinical responses, we conducted a Wilcoxon signed‐rank analysis on the connectivity between contacts eliciting specific clinical responses, compared to the connectivity between contacts eliciting different responses. To minimize the influence of contact distance, we calculated the Euclidean distance of the contacts, and the connectivity matrix was transformed using the Yeo‐Johnson transformation prior to statistical analysis.

## Author Contributions

L.R. conceived and designed the research. L.R., D.W., C.X., S.W., L.Q., G.J., X.W., Y.C., J.D., D.H., J.G., Y.Z., Y.W., H.Z., Y.D., H.Y., R.G., Z.Z., and P.W. conducted the research and investigation process. C.X., T.Y., X.W., Y.W., H.Z., Y.D., R.G., Y.G., and P.W. provided the study materials. D.W., C.X., L.Q., and G.J. wrote the draft. Y.W., P.K., F.B., T.Y., L.R., and G.Z. revised the manuscript. T.Y., L.R., and G.Z. supervised the study. All authors have read and approved the final manuscript.

## Funding

This work was supported by the Beijing Research Ward Excellence Program (BRWEP2024W022010200), the National Natural Science Foundation of China (82371457), the Beijing Hospitals Authority Clinical Medicine Development of Special Funding Support (ZLRK202321), the National Key R & D Program of China (2022YFC2503805, 2024YFA1306903), and the Natural Science Foundation of Shandong Province (ZR2024MH123), all awarded to Liankun Ren.

## Ethics Statement

The study was conducted according to the guidelines of the Declaration of Helsinki and was approved by the Ethics Committee of Xuanwu Hospital, Capital Medical University ([2024]126). Each patient representative voluntarily provided written informed consent to participate in this study.

## Conflicts of Interest

The authors declare no conflicts of interest.

## Supporting information




**Supporting Information file 1**: mco270931‐sup‐0001‐TableS1.xlsx.

**Supporting Information file 2**: mco270931‐sup‐0002‐TableS2.docx.

## Data Availability

The data are not publicly available as they contain information that could compromise research participant privacy or consent. The data are available from the corresponding author upon reasonable request. The toolbox used for analyzing the functional connectivity is available within the Lead‐DBS software (www.lead‐dbs.org). Code used to analyze the dataset is openly available within the Lead‐DBS/‐Connectome software.
