# Supplementary material for: Unveiling Functional Architecture of Human Thalamus: AStereoelectroencephalography Study
Source: MedComm (2020). 2026 Sep 2;7(9):e70931. doi: 10.1002/mco2.70931 (PMC13537012; doi:10.1002/mco2.70931)
Supplement: Supplementary file 1 [file MCO2-7-e70931-s002.docx]

Supplementary file

for

**Unveiling Functional Architecture of Human Thalamus: a Stereoelectroencephalography Study**

Liankun Ren^1,2,3, #*^, Di Wu^1,2,3,#^, Cuiping Xu^3,4,#^, Lei Qi^5,^ , Guangyuan Jin^1,2,3,^ , Xueyuan Wang^3,4^, Yueyang Cheng^1,2,3^, Jialin Du^6^, Siyi Wang^1,2,3,^, Dongmei Hu^1,2,3^, Junliang Ge^1,2,3^, Yixi Zheng^1,2,3^, Yihe Wang^4^, Huaqiang Zhang^4^, Yang Dai^4^, Hao Yan^4^, Runshi Gao^4^, Ying Gao^4^, Zeyu Zhang^7^, Penghu Wei^4^, Yuping Wang^1,2,3,8^, Philippe Kahane^9^, Fabrice Bartolomei^10,11^, Tao Yu^3,4,*^, and Guoguang Zhao^2,3,4,*^

^1^ Department of Neurology, Xuanwu Hospital, Capital Medical University, Beijing, China,

^2^ Clinical Research Center of Epilepsy, Xuanwu Hospital, Capital Medical University, Beijing, China,

^3^ National Center for Neurological Disorders, Beijing, China,

^4^ Department of Neurosurgery, Xuanwu Hospital, Capital Medical University, Beijing, China,

^5^ Department of Neurology, China rehabilitation research center, Beijing, China,

^6^ Department of Pharmacy Phase I Clinical Trial Center, Xuanwu Hospital, Capital Medical University, Beijing, China,

^7^ Department of science, the University of Melbourne, Victoria, Australia,

^8^ Institute of sleep and consciousness disorders, Center of Epilepsy, Beijing Institute for Brain Disorders, Capital Medical University, Beijing, China,

^9^ Grenoble Alpes University Hospital Center, Grenoble Alpes University, Inserm, U1216, Grenoble Institute Neurosciences, Grenoble, France,

^10^ Aix Marseille Univ, INSERM, INS, Inst Neurosci Syst, Marseille, France,

^11^ Epilepsy and Clinical Neurophysiology Department, Timone Hospital, Assistance publique-Hôpitaux de Marseille, Marseille, France,

^#^ These authors contributed equally to this work.

*Corresponding author: Tao Yu, Liankun Ren, and Guoguang Zhao

Corresponding author’s address: Xuanwu Hospital, Capital Medical University, 45 Changchun Street, 100053 Beijing, China.

Corresponding author’s phone and fax: 010-83198899

Corresponding author’s e-mail address: yutaoly@sina.com (Tao Yu), renlk2022@outlook.com (Liankun Ren), ggzhao@vip.sina.com (Guoguang Zhao)

**Table S2. Thalamus subgroups divided by the DISTAL atlas**

| **Subdivision group** | **Constituent parts** |
| --- | --- |
| Anterior group | Nucleus Anterventralis |
|  | Lateral dorsal Nucleus |
| Medial group | Medioventral Nucleus |
|  | Mediodorsal Nucleus |
|  | Central Median Nucleus |
|  | Nucleus parafascicularis |
| Lateral group | Ventral Anterior Nucleus (Lpo) |
|  | Ventral Anterior Nucleus (D.o.e) |
|  | Basal ventral medial nucleus/Ventral posterior inferior nucleus |
|  | Ventral Medial Nucleus |
|  | Ventral Lateral Posterior Nucleus (V.o.i VLp) |
|  | Ventral Lateral Anterior Nucleus (V.o.a) |
|  | Ventral Lateral Anterior Nucleus (V.o.p VLa) |
|  | Ventral Anterior Nucleus (D.o.i VA) |
|  | Ventral Anterior Nucleus (Z.o.) |
|  | Ventral Lateral Posterior Nucleus (V.im.i) |
|  | Ventral Lateral Posterior Nucleus (Z.im.e) |
|  | Ventral Lateral Posterior Nucleus (Z.im.i) |
|  | Ventral Lateral Posterior Nucleus (V.im.e VLp) |
|  | Ventral Posterior Medial Nucleus (V.c.i) |
|  | Ventral Posterior Lateral Nucleus (V.c.a.i VPLa) |
|  | Ventral Posterior Lateral Nucleus (Z.c.e) |
|  | Ventral Posterior Lateral Nucleus (Z.c.i) |
|  | Ventral Lateral Posterior Nucleus (V.im.i) + |
|  | Ventral Lateral Posterior Nucleus (D.im.i) |
|  | Ventral Lateral Posterior Nucleus (D.im.e) |
|  | Ventral Posterior Medial Nucleus (V.c.a.i VPM) |
|  | Ventral Lateral Posterior Nucleus (Z.im) |
|  | Ventral Posterior Lateral Nucleus (V.c.e) |
|  | Ventral Posterior Inferior Nucleus |
|  | Ventral Posterior Lateral Nucleus (V.c.p.e) |
|  | Ventral Lateral Posterior Nucleus (V.o.p VLp) |
|  | Ventral Lateral Anterior Nucleus (Z.im.e) |
|  | Ventral Lateral Anterior Nucleus (V.im.e VLa) |
|  | Ventral Lateral Anterior Nucleus (D.o.i VLa) |
| Posterior group | Posterior nucleus |
|  | Lateral Posterior Nucleus (D.c) |
|  | Lateral Posterior Nucleus (Pu.o.l) |
|  | Lateral Posterior Nucleus (Pu.o.m) |
|  | Anterior Pulvinal Nucleus (V.c.por) |
|  | Anterior Pulvinal Nucleus (Pu.o.v) |
|  | Inferior Pulvinar Nucleus |
|  | Medial Pulvinar Nucleus (Pu.m) |
|  | Lateral Pulvinar Nucleus |
|  | Nucleus pulvinaris superficialis |
|  | Medial Pulvinar Nucleus (Pu.m) + |
